# Validation of evaluating left ventricular diastolic function with estimated left atrial volume from anteroposterior diameter

**DOI:** 10.1186/s12872-021-01920-y

**Published:** 2021-02-23

**Authors:** Yonghuai Wang, Liang Zhang, Shuang Liu, Guangyuan Li, Fanxin Kong, Cuiting Zhao, Jun Yang, Chunyan Ma

**Affiliations:** Department of Cardiovascular Ultrasound, The First Hospital of China Medical University, No. 155 Nanjingbei Street, Shenyang, 110001 Liaoning People’s Republic of China

**Keywords:** Diastolic function, Left atrial volume, Anteroposterior diameter, Echocardiography, Recommendations

## Abstract

**Background:**

Left atrial (LA) volume (LAV) is one of the recommended key variables for evaluating left ventricular (LV) diastolic function. However, only LA anteroposterior diameter (LAAP) is available in numerous large-scale existing databases. Therefore, this study aimed to validate whether LV diastolic function could be evaluated with estimated LAV from LAAP.

**Methods:**

A total of 552 inpatients with sinus rhythm were consecutively enrolled. LAV was measured by biplane Simpson’s disk summation method. LV diastolic function was evaluated according to the 2016 proposed recommendations. Best-fitting regression models of LAAP index (LAAPI)–LAV index (LAVI) were developed and equations with the highest *F*-value were chosen in the first 276 subjects (derivation set), and concordance for evaluating LV diastolic function between using estimated and observed LAVI was verified in the remaining 276 subjects (validation set).

**Results:**

In the derivation set, the linear model has the highest *F*-value in all subjects and in the subjects with normal or depressed LV ejection fraction. In the validation set, using the linear equation (LAVI = 2.05 × LAAPI − 13.86), the higher area under curve and narrower range of difference were shown between estimated LAVI and observed LAVI, respectively. Further, concordance for diagnosis (overall proportion of agreement, 88.4%; *κ* = 0.79) and grading (overall proportion of agreement, 84.8%; *κ* = 0.74) of LV diastolic dysfunction was substantial between using estimated and observed LAVI.

**Conclusions:**

LV diastolic function can be evaluated with estimated LAVI from LAAPI, which might provide a surrogate method when the direct measurement of LAV is not available.

## Introduction

Left ventricular (LV) diastolic dysfunction (DD) is associated with an increase in all-cause mortality in the general population, even in the preclinical stage [1]. The presence of LVDD is a cardinal feature to diagnose heart failure with preserved LV ejection fraction (LVEF) [2, 3]. Therefore, an accurate evaluation of LV diastolic function (DF) is of pivotal importance in routine clinical settings.

Recently, the proposed recommendations by the American Society of Echocardiography (ASE) and the European Association of Cardiovascular Imaging (EACVI) in 2016 integrated two-dimensional echocardiographic and Doppler echocardiographic parameters and provided a practical and reliable algorithm for diagnosing and grading LVDD irrespective of LVEF [4]. Among these parameters, left atrial (LA) volume index (LAVI) is the recommended key two-dimensional variable for evaluating LVDF. However, LAV is not available in numerous early study designs, such as the preliminary stage of Framingham Heart Study [5, 6], the Strong Heart Study [7], the Jackson Heart Study [8], and others, or even in several current large-scale cross-sectional analysis [9], which has precluded retrospective analyses of large-scale existing databases related to LVDF.

Although the LAV measured using the biplane disk summation algorithm is more accurate and recommended to describe LA size in routine clinical practice and research [10, 11], LA anteroposterior diameter (LAAP) is present extensively in plenty of large-scale databases or trials because it is relatively more readily available and has better measurement reproducibility than LAV [12–14]. However, whether LAVI can be estimated from LAAP index (LAAPI) and then be used for evaluating LVDF when the direct measurement of LAV is no available is still not well understood.

In view of the foregoing considerations, we conducted this study to develop the best-fitting regression models and seek the optimum equations for estimating LAVI from LAAPI, and validate whether LVDF could be evaluated with estimated LAVI, which might provide a surrogate method to evaluate LVDF when the direct measurement of LAV is not available or the LAV values are highly variable.

## Material and methods

### Study population

We prospectively and consecutively enrolled the inpatients in sinus rhythm from the Cardiology Department of our hospital from January 2019 to October 2019.

In accordance with the 2016 recommendations for the evaluation of LVDF from the ASE and EACVI [4], patients with specific cardiovascular disease in which the evaluating algorithm had significant limitations were excluded, such as the patients with atrial fibrillation, sinus tachycardia, atrioventricular block, left bundle branch block, hypertrophic and restrictive cardiomyopathy, significant mitral annular calcification, mitral stenosis, moderate and severe mitral regurgitation, severe aortic regurgitation, noncardiac pulmonary hypertension, right ventricular pacing, cardiac resynchronization therapy, valvular heart surgery or intervention and heart transplantation. Moreover, to obtain accurate measurements of LAV and LAAP, patients with poor quality of echocardiography, hyper-mobile interatrial septum or interatrial septal aneurysm, dilated aortic root or ascending aortic encroaching on the LA anterior wall, dilated or tortuous descending aorta indenting the LA posterior wall, and severe calcification at aortic sinuses and aortic valve were also excluded from this study.

A total of 552 subjects (mean age 58.4 ± 11.1 years, 221 women) met the eligibility criteria during the study period. These subjects were randomly divided into two groups: (1) derivation set (n = 276, mean age 59.1 ± 10.8 years, 114 women), in which best-fitting regression models and equations of LAVI–LAAPI were developed; and (2) validation set (n = 276, mean age 57.7 ± 11.3 years, 107 women), in which concordance for evaluating LVDF between using estimated and observed LAVI was verified.

Written informed consent was obtained from all patients before enrollment. The study protocol was approved by the China Medical University Ethics Committee and was conducted in line with the ethical guidelines of the 1975 Declaration of Helsinki.

### Echocardiography

All subjects were examined at rest using a Vivid E9 ultrasound system (GE Healthcare, Waukesha, WI, USA) equipped with M5S phased-array probe. Standard two-dimensional cine loops, including at least 3 consecutive cardiac cycles, and Doppler spectrum during normal respiration (< 20 breaths/min) were recorded for offline analysis using an EchoPAC workstation (GE Healthcare). Both image acquisition and quantitative analysis were performed according to the recommendations of transthoracic echocardiographic examination and cardiac chamber quantification from ASE by two experienced cardiologists who were blinded to any clinical data [15, 16].

**Fig. 1:**
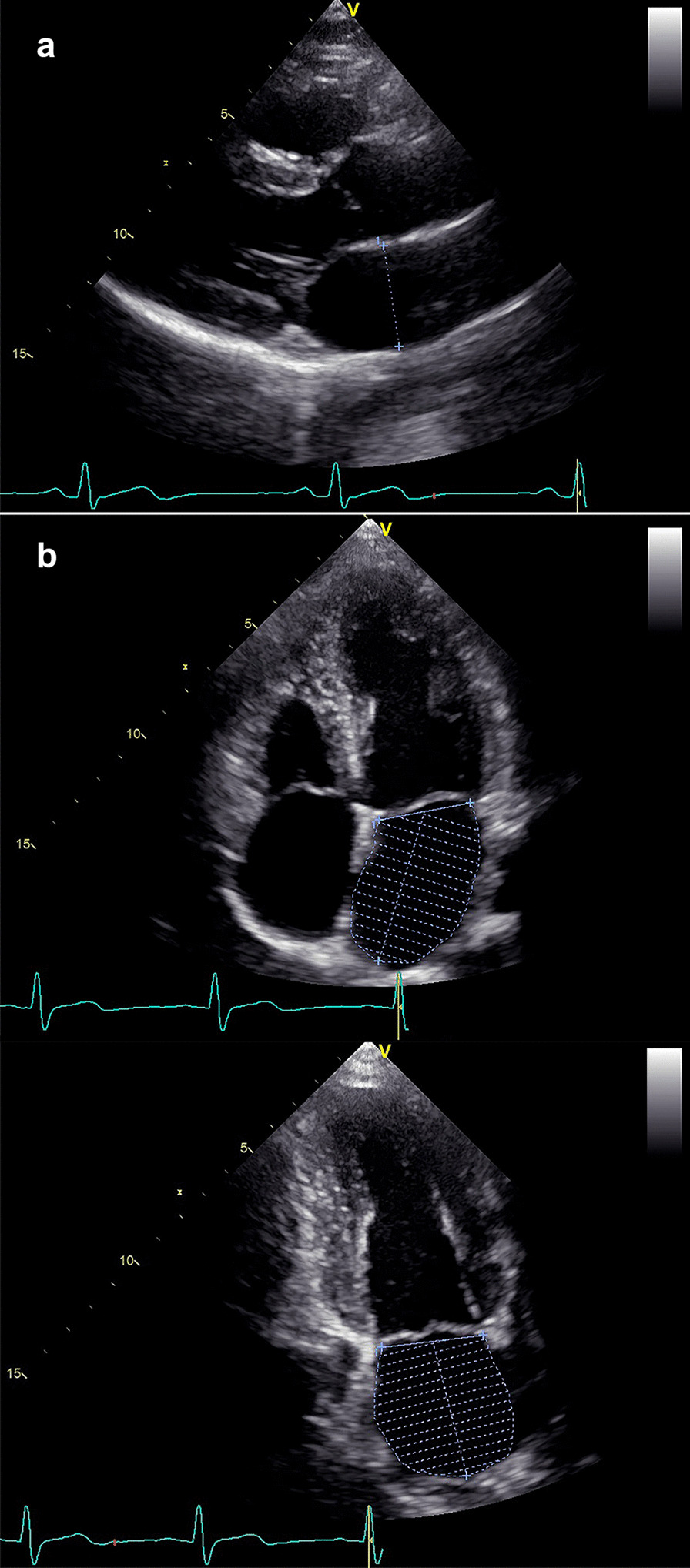
Measurement methods of LAAP (**a**) and LAV (**b**). *LAAP* left atrial anteroposterior diameter, *LAV* left atrial volume

LVEF was measured using the biplane Simpson’s disk summation method from both the apical two- and four-chamber views during LV end-diastole. LVEF < 52% in males or < 54% in females was considered depressed [15]. LAAP was measured during LV end-systole from the parasternal long-axis view [15]. LAV was measured using the biplane Simpson’s disk summation method from both the apical two- and four-chamber views during LV end-systole (Fig. 1). LAVI and LAAPI were LAV and LAAP divided by body surface area, respectively. Mitral inflow early diastolic (E) and late diastolic (A) peak velocity by pulsed flow Doppler, the septal and lateral early diastolic mitral annulus peak velocity (e′) by pulsed tissue Doppler, and tricuspid regurgitation (TR) systolic jet velocity by continuous Doppler were measured in the apical four-chamber views. Then E/A, average e′ and average E/e′ were calculated.

The diagnosis and grading of LVDD was performed according to 2016 ASE/EACVI recommendations [4]. For patients with normal LVEF, four variables were recommended for diagnosing LVDD (septal e′ < 7 cm/s or lateral e′ < 10 cm/s, average E/e′ > 14, LAVI > 34 mL/m^2^, TR velocity > 2.8 m/s). Patients were diagnosed with normal DF if none or only one variable met the cutoff values, DD if more than two of the variables met the cutoff values, and indeterminate if just two variables met the cutoff values. In patients with normal LVEF and myocardial disease or DD, and in those with depressed LVEF, grade I DD was diagnosed if E/A ratio ≤ 0.8 and E ≤ 50 cm/s and grade III DD if E/A ratio ≥ 2. If E/A ≤ 0.8 and E > 50 cm/s, or 0.8 < E/A < 2, additional variables are needed (average E/e′ > 14, LAVI > 34 mL/m^2^, TR velocity > 2.8 m/s). Grade I DD was diagnosed if only one of the variables met the cutoff values, and grade II DD if more than two of the variables met the cutoff values. However, if only one variable is available, or only two variables are available but discrepant, LV diastolic dysfunction grade could not be determined.

### Reproducibility

Inter- and intra-observer agreement of LAAP and LAV measurements were examined in 10 randomly selected patients. To assess intra-observer variability, the same doctor repeated the measurements after > 4 weeks. In addition, to assess inter-observer variability, the two doctors independently repeated the measurements twice.

### Statistical analysis

Statistical analyses were performed using SPSS version 21.0 statistical software (IBM Corp, Armonk, NY) and R version 3.5.1 (R Foundation for Statistical Computing, Vienna, Austria). Normality plots with tests were performed using the Shapiro–Wilk test. Continuous data were expressed as mean ± standard deviation (SD) or median (interquartile range), and categorical variables as numbers and percentages. In the comparison between derivation set and validation set, differences in continuous variables were assessed by independent samples t-test or Mann–Whitney U test where appropriate, and categorical variables by chi-square test or Fisher exact test. Correlation between LAVI and LAAPI was sought using Pearson correlation analyses. In the derivation set, the best-fitting regression models between LAVI and LAAPI were developed, including linear, logarithmic, quadratic, cubic, compound, power, S, growth, and exponential. The *F*-values, which express the statistical significance of the different models, of these models were compared. The regression equations with the highest *F*-values were chosen to calculate the estimated LAVI in the validation set. The comparison between the observed LAVI with estimated LAVI by each equation was performed by paired samples t-test or Wilcoxon test, where appropriate. The variability of estimated and observed LAVI was assessed by calculating the intra-class correlation coefficient and 95% confidence interval (CI) using a two-way random-effects model. Receiver operating characteristic (ROC) curve analysis and area under curve (AUC) were used to evaluate the performance for identifying observed LAVI > 34 mL/m^2^. Bland–Altman analyses were used for assessing the bias and limits of agreement (LOA) between the observed and estimated LAVI, and estimating the inter-observer and intra-observer reproducibility of LAAP and LAV. The concordance for evaluating LV diastolic function between using estimated and observed LAVI were tested by calculating the *κ* coefficient and the overall proportion of agreement (proportion of subjects identically classified). The calculated *κ* coefficient was judged as follows: 0 ≤ *κ* < 0.2 slight; 0.2 ≤ *κ* < 0.4 fair; 0.4 ≤ *κ* < 0.6 moderate; 0.6 ≤ *κ* < 0.8 substantial, and 0.8 ≤ *κ* < 1 perfect [17].

## Results

The demographic characteristics and echocardiographic measurements of the population in the derivation and validation sets are listed in Table 1. There were no significant differences in age, sex, body surface area, and heart rate between the groups (*P* > 0.05 for all). There were similar prevalence rates between the groups in depressed LVEF, LAVI > 34 mL/m^2^, septal e′ velocity < 7 cm/s, lateral e′ velocity < 10 cm/s, average E/e′ > 14, and TR velocity > 2.8 m/s (P > 0.05 for all).

**Table 1:** Demographic characteristics and echocardiographic measurements of the population

Variable	Derivation set (n = 276)	Validation set (n = 276)	*P* value
Age (years)	59.1 ± 10.8	57.7 ± 11.3	0.13
Female [n (%)]	114 (41.3%)	107 (38.8%)	0.52
Body surface area (m^2^)	1.76 ± 0.17	1.78 ± 0.18	0.40
Heart rate (bpm)	69.1 ± 12.5	69.7 ± 11.9	0.60
LV end-diastolic diameter (mm)	53.11 ± 8.74	52.99 ± 8.54	0.87
LV end-diastolic volume (mL)	94.00 (78.00–122.00)	97.00 (79.25–127.00)	0.69
LVEF (%)	56.69 ± 12.12	56.86 ± 12.13	0.87
Depressed LVEF [n (%)]	59 (21.4%)	60 (21.7%)	0.92
LAAP (mm)	39.44 ± 5.94	39.25 ± 6.21	0.72
LAAPI (mm/m^2^)	22.50 ± 3.68	22.20 ± 3.49	0.32
LAAPI > 23 mm/m^2^ [n (%)]	105 (38.0%)	89 (32.2%)	0.15
LAV (mL)	53.00 (43.00–66.83)	51.50 (42.00–67.00)	0.62
LAVI (mL/m^2^)	30.19 (24.16–36.73)	29.03 (23.59–37.13)	0.48
LAVI > 34 mL/m^2^ [n (%)]	94 (34.1%)	89 (32.2%)	0.65
Septal e′ velocity (cm/s)	6.09 ± 2.30	6.11 ± 2.49	0.92
Septal e′ velocity < 7 cm/s [n (%)]	168 (60.9%)	168 (60.9%)	1.00
Lateral e′ velocity (cm/s)	8.88 ± 3.31	8.70 ± 3.40	0.51
Lateral e′ velocity < 10 cm/s [n (%)]	163 (59.1%)	174 (63.0%)	0.36
Average E/e′	10.96 ± 5.54	11.24 ± 5.74	0.56
Average E/e′ > 14 [n (%)]	51 (18.5%)	54 (19.6%)	0.78
TR velocity > 2.8 m/s [n (%)]	37/214	35/229	0.57

Values shown are mean ± SD, median (interquartile range), or frequency (percentages)

*LV* left ventricle, *LVEF* left ventricular ejection fraction, *LAAP* left atrial anteroposterior diameter, *LAAPI* left atrial anteroposterior diameter index, *LAV* left atrial volume, *LAVI* left atrial volume index, *e′* early diastolic annular peak velocity, *E* early diastolic flow peak velocity, *TR* tricuspid regurgitation

### Analysis in derivation set

**Fig. 2:**
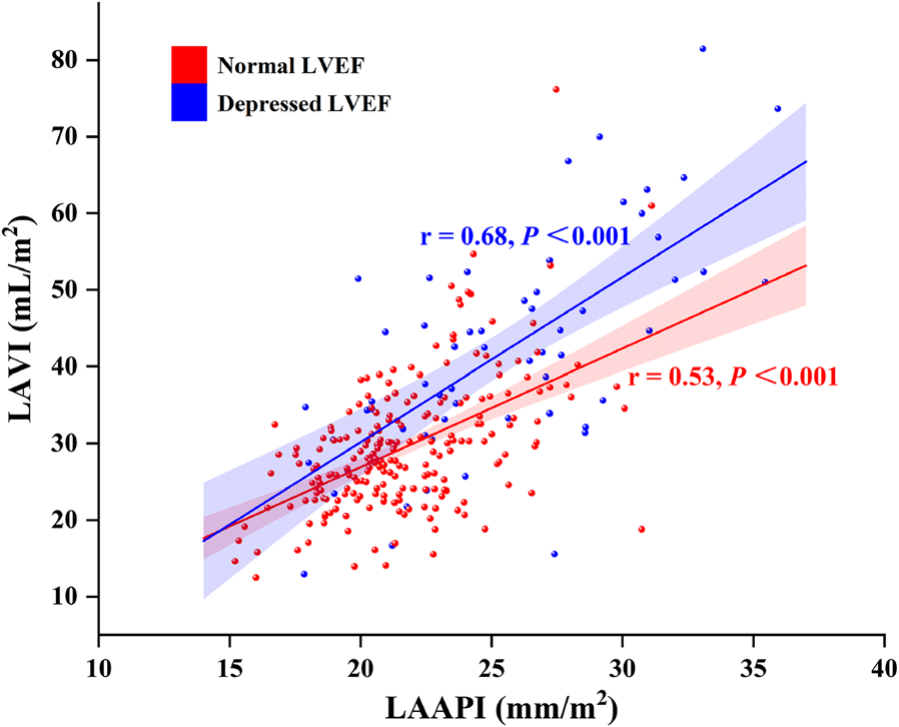
Correlations between LAVI and LAAPI according to LVEF. *LAVI* left atrial volume index, *LAAPI* left atrial anteroposterior diameter index, *LVEF* left ventricular ejection fraction

There was a significant correlation between LAVI and LAAPI (r = 0.67, *P* < 0.001), and the correlation coefficient between LAVI and LAAPI in the subjects with depressed LVEF (r = 0.68, *P* < 0.001) was larger than that in subjects with normal LVEF (r = 0.53, *P* < 0.001) (Fig. 2).

Table 2 displays the best-fitting regression models for computation of LAVI from LAAPI in derivation set. The linear model had the highest *F*-value in all subjects (*r*^*2*^ = 0.44, *F* = 218.60, *P* < 0.001) and in the subjects with normal or depressed LVEF (*r*^*2*^ = 0.28, *F* = 83.06, *P* < 0.001; *r*^*2*^ = 0.46, *F* = 48.65, *P* < 0.001). Therefore, we estimated LAVI from LAAPI stratified by LVEF using two regression equations (first equation: LAVI = 2.05 × LAAPI − 13.86 in all subjects; second equation: LAVI = 1.54 × LAAPI − 3.97 in the subjects with normal LVEF and LAVI = 2.15 × LAAPI − 12.83 in the subjects with depressed LVEF).

**Table 2:** Best-fitting regression models for computation of LAVI from LAAPI in the derivation set

Equation	r^2^	*F*	*P* value	Constant	β1	β2	β3
All subjects (n = 276)							
Linear	**0.44**	**218.60**	< 0.001	− 13.86	2.05		
Logarithmic	0.42	202.20	< 0.001	− 112.93	46.80		
Quadratic	0.45	113.70	< 0.001	19.75	− 0.82	0.06	
Cubic	0.45	113.83	< 0.001	13.77	0.00	0.02	0.0005
Compound	0.40	182.78	< 0.001	8.55	1.06		
Power	0.40	181.13	< 0.001	0.51	1.32		
S	0.39	173.69	< 0.001	4.75	− 29.29		
Growth	0.40	182.78	< 0.001	2.15	0.06		
Exponential	0.40	182.78	< 0.001	8.55	0.06		
Subjects with normal LVEF (n = 217)							
Linear	**0.28**	**83.06**	< 0.001	− 3.97	1.54		
Logarithmic	0.28	82.80	< 0.001	− 74.53	33.92		
Quadratic	0.28	41.42	< 0.001	− 10.77	2.16	− 0.01	
Cubic	0.28	41.43	< 0.001	− 8.77	1.87	0.00	− 0.0002
Compound	0.27	78.31	< 0.001	9.84	1.05		
Power	0.27	81.37	< 0.001	1.00	1.09		
S	0.28	82.61	< 0.001	4.45	− 23.44		
Growth	0.27	78.31	< 0.001	2.29	0.05		
Exponential	0.27	78.31	< 0.001	9.84	0.05		
Subjects with depressed LVEF (n = 59)							
Linear	**0.46**	**48.65**	< 0.001	− 12.83	2.15		
Logarithmic	0.45	45.92	< 0.001	− 131.36	53.79		
Quadratic	0.47	24.53	< 0.001	20.51	− 0.48	0.05	
Cubic	0.47	24.53	< 0.001	16.62	0.00	0.03	0.0002
Compound	0.40	38.25	< 0.001	10.53	1.05		
Power	0.40	38.10	< 0.001	0.57	1.31		
S	0.39	37.00	< 0.001	4.97	− 32.05		
Growth	0.40	38.25	< 0.001	2.35	0.05		
Exponential	0.40	38.25	< 0.001	10.53	0.05		

The bold values indicated the selected regression model for computation of LAVI from LAAPI

*LVEF* left ventricular ejection fraction, *LAAPI* left atrial anteroposterior diameter index, *LAVI* left atrial volume index

### Analysis in validation set

Before estimating LAVI, we first analyzed the identifying performance of LAAPI for LAVI > 34 mL/m^2^ [AUC, 0.85 (0.80–0.90);* P* < 0.001], and the concordance between LAAPI > 23 mm/m^2^ with LAVI > 34 mL/m^2^ (overall proportion of agreement, 77.0%; *κ* = 0.47).

**Fig. 3:**
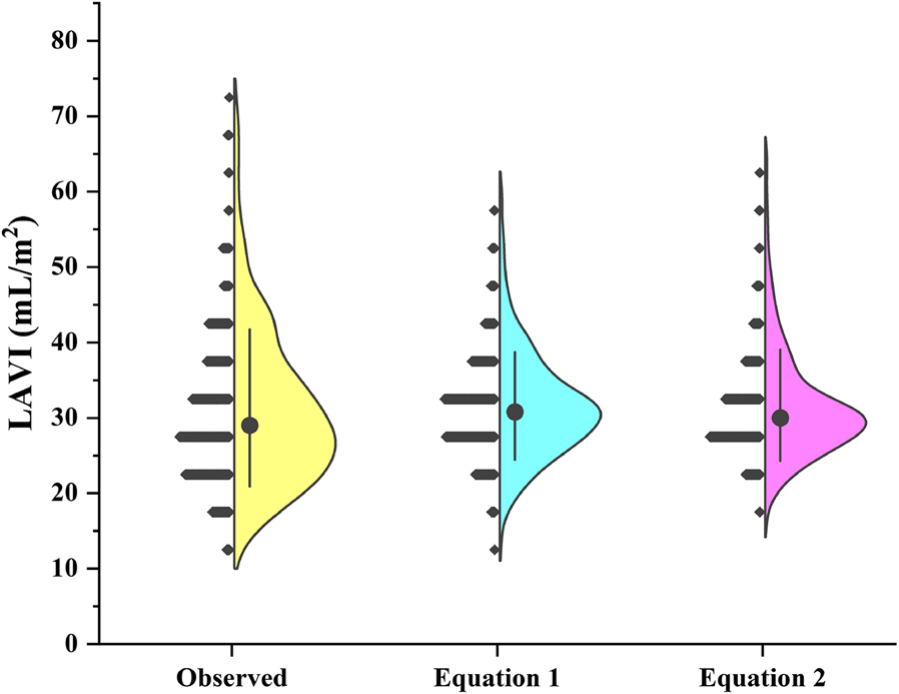
Comparison of the observed LAVI with estimated LAVI. *LAVI* left atrial volume index

After estimating LAVI using the abovementioned two regression equations in the validation set, we compared the observed LAVI with estimated LAVI by each regression equation respectively, and found that there were no significant differences between the observed LAVI [29.03 (23.59–37.13) mL/m^2^] and estimated LAVI by the first equation [30.79 (26.88–35.42) mL/m^2^] and the second equation [29.98 (27.23–33.98) mL/m^2^] (Fig. 3).

**Fig. 4:**
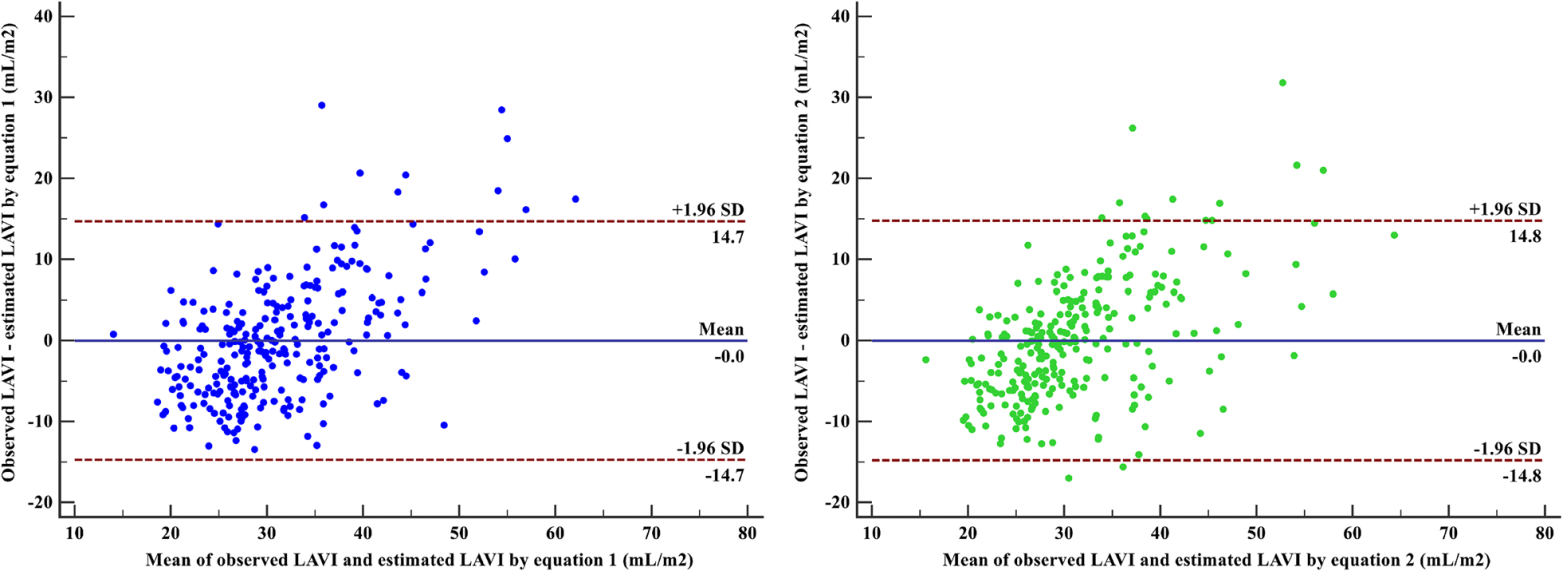
Bland–Altman analyses of the observed and estimated LAVI. *LAVI* left atrial volume index

Table 3 displays the intra-class correlations between estimated and observed LAVI, identifying performance of estimated LAVI for observed LAVI > 34 mL/m^2^, and concordance between estimated and observed LAVI > 34 mL/m^2^, respectively. The estimated LAVI using the first equation showed the similar intra-class correlations coefficient [*ρ*, 0.63 (0.55–0.70); *P* < 0.001] and overall proportion of agreement (78.98%) with observed LAVI, but the slightly higher AUC [0.85 (0.80–0.90);* P* < 0.001] for identifying observed LAVI > 34 mL/m^2^. In Bland–Altman analysis, the estimated LAVI using the first equation showed the slightly narrower range of difference compared with observed LAVI (Fig. 4). Therefore, we finally used the first equation to estimate LAVI from LAAPI and further evaluate LVDF.

**Table 3:** Relationship between observed and estimated LAVI in the validation set

	Equation 1	Equation 2
Intra-class correlations^a^		
Coefficient (95% CI)	0.63 (0.55–0.70)	0.63 (0.55–0.70)
*P* value	< 0.001	< 0.001
ROC analyses^b^		
Area under curve (95% CI)	0.85 (0.80–0.90)	0.84 (0.79–0.89)
*P* value	< 0.001	< 0.001
Concordance test^c^		
*κ* coefficient	0.50	0.49
Overall proportion of agreement	78.98%	78.98%
Bland–Altman analyses		
Mean difference	− 0.02	− 0.02
Limits of agreement	− 14.73 to 14.68	− 14.80 to 14.76

*LAVI* left atrial volume index, *ROC* receiver operating characteristic, *CI* confidence interval

^a^Intra-class correlations between observed and estimated LAVI

^b^Identifying performance of estimated LAVI for observed LAVI > 34 mL/m^2^

^c^Concordance between observed and estimated > 34 mL/m^2^

**Fig. 5:**
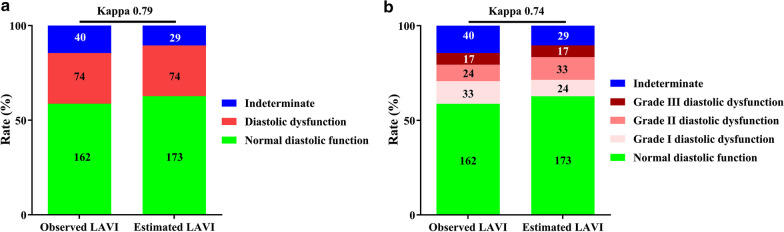
Concordance for diagnosis (**a**) and grading (**b**) of LVDD between using observed and estimated LAVI. *LVDD* left ventricular diastolic dysfunction, *LAVI* left atrial volume index

Concordance for diagnosis (overall proportion of agreement, 88.4%; *κ* = 0.79) and grading (overall proportion of agreement, 84.8%; *κ* = 0.74) of LVDD was substantial between using estimated and observed LAVI (Table 4 and Fig. 5). The overall reclassification rate was 15.2% (42 patients). Seven patients (2.5%) had normal DF according to observed LAVI but indeterminate on the basis of estimated LAVI. One patient (0.4%) and 9 patients (3.3%) with grade I DD according to observed LAVI were reclassified to normal DF and grade II DD using estimated LAVI, respectively. One patient (0.4%) and 3 patients (1.1%) with grade II DD according to observed LAVI were reclassified to grade I DD and indeterminate according to estimated LAVI. And Seventeen patients (6.2%) and 4 patients (1.4%) with indeterminate according to observed LAVI were reclassified to normal DF and grade II DD using estimated LAVI, respectively.

**Table 4:** Reclassification cross-relations for diagnosis and grading of LV DD between using estimated and observed LAVI

	Using observed LAVI					
	Normal DF	Grade I DD	Grade II DD	Grade III DD	Indeterminate	Total
Using estimated LAVI						
Normal DF	155	1	0	0	17	173
Grade I DD	0	23	1	0	0	24
Grade II DD	0	9	20	0	4	33
Grade III DD	0	0	0	17	0	17
Indeterminate	7	0	3	0	19	29
Total	162	33	24	17	40	276

*LV* left ventricle, *DD* diastolic dysfunction, *LAVI* left atrial volume index, *DF* diastolic function

### Reproducibility

Inter-observer and intra-observer reproducibility for LAAP and LAV was high. The inter-observer reproducibility for LAAP and LAV had a bias of 0.5 mm (LOA, − 0.5 to 1.4 mm), and 0.6 mL (LOA, − 5.3 to 6.4 mL), respectively. The intra-observer reproducibility for LAAP and LAV had a bias of − 0.1 mm (LOA, − 1.1 to 0.9 mm), and 0.6 mL (LOA, − 4.7 to 5.8 mL), respectively.

## Discussion

In the present study, we developed the best-fitting regression models for estimating LAVI from LAAPI and established the optimum linear regression equations in the subjects with normal LVEF and depressed LVEF in the derivation set, respectively. We also validated that LVDF could be evaluated with estimated LAVI from LAAPI in the validation set when the direct measurement of LAV is not available or the LAV values are highly variable.

Normal LVDF plays a significant role in ensuring normal LV filling and adequate cardiac output. LVDD has been recognized to be associated with incident heart failure and resultant poor outcomes [18, 19], and even related to a marked increase of all-cause mortality in the general population [1]. Therefore, the accurate evaluation of LVDF is increasingly becoming an essential element in routine clinical settings. Among imaging techniques to evaluate LVDF, echocardiography has become a widely used and powerful tool for diagnosing LVDD as well as further tracking of pathological changes and even making clinical decision on a day-to-day basis [20, 21].

LA size is one of the recommended indispensable elements for evaluating LVDF by echocardiography as per recommendations from ASE and EACVI because it can reflect the chronicity and severity of LVDD [4]. To describe LA size, LAV is the preferred method over LA diameter since LA dilation in each dimension may not be uniform due to the physical confinement of the adjacent structure [22, 23]. That notwithstanding, LAAP is widely used in routine practice or a multicenter setting, and is present extensively in numerous large databases or trials because it is relatively more readily available and has better measurement reproducibility than LAV [24, 25]. In this study, we validated that LVDF also could be evaluated with estimated LAVI from LAAPI when the direct measurement of LAV is not available. However, our most noteworthy objective is to provide a surrogate to evaluate LVDF when the directly measured LAV is not available on retrospective analysis of the older existing large-scale databases, rather than directly substituting the recommended algorithm for diagnosing and grading of LVDD from ASE and EACVI.

Maybe, it was an optimistic thinking that LAAPI > 23 mm/m^2^ could be directly substituted as a variable for LAVI > 34 mL/m^2^ to assess LV DF when directly measured LAV is not available. However, our study revealed that the concordance between LAAPI > 23 mm/m^2^ with LAVI > 34 mL/m^2^ were lower than the estimated LAVI obtained by the proposed regression equation in our study. Therefore, our results indicated that this optimistic idea was not recommended when directly measured LAV is unavailable.

Recently, Canciello et al. [26] developed regression models to compute LAV by the biplane Simpson’s disk summation method, area-length method, and elliptical method from LAAP respectively in the derivation series including 70 subjects and reported that LAV by elliptical method can be predicted with good accuracy by simple measurement of LAAP using a nonlinear equation. However, the elliptical method may be more susceptible to geometric assumptions about LA shape and thus may underestimate the actual LAVI and hinder the accurate definition of LA dilatation [27, 28]. Notably, our study established the optimum regression equations for estimating LAVI by biplane Simpson’s disk summation method, as recommended by ASE and EACVI in routine clinical practice, in a larger sample size. Moreover, our study further validated that the estimated LAVI could also be used to evaluate LVDF, which might provide a surrogate method to evaluate LVDF when the direct measurement of LAV is not available or the LAV values are highly variable. Because the ASE recommendation is universal, our results may also be applicable for the other ethnicities and races, and may also provide a valuable reference for the clinicians in different regions, although our study was based on the Chinese population whose LA size may be different from other ethnic or racial populations.

### Limitations

The major limitation of our study was related to the exclusion of the patients with several specific cardiovascular diseases, such as atrial fibrillation, mitral valve dysfunction, left bundle branch block, hypertrophic cardiomyopathy, and so on, in which the evaluation of LVDF required the determination of additional specific parameters according to the 2016 recommendations [4]. This may limit the generalizability of our findings in these patients.

Another study limitation was the relatively low percentage of patients with depressed LVEF and limited range of LAVI, which was mainly attributable to the unselected and consecutively recruited population for reducing selection bias in the present study, and a multicenter study with a larger number of patients with depressed LVEF and a wide range of LAVI should be designed to verify the findings.

## Conclusions

LVDF can be evaluated with estimated LAVI from LAAPI, which might provide a surrogate method when the direct measurement of LAV is not available or the LAV values are highly variable. It is necessary to verify these results in future practice.

## Data Availability

The datasets during and/or analyzed during the current study is available from the corresponding author on reasonable request.

## Abbreviations

LV: Left ventricular
DD: Diastolic dysfunction
LVEF: Left ventricular ejection fraction
DF: Diastolic function
ASE: American Society of Echocardiography
EACVI: European Association of Cardiovascular Imaging
LA: Left atrial
LAV: Left atrial volume
LAAP: Left atrial anteroposterior diameter
TR: Tricuspid regurgitation
SD: Standard deviation
CI: Confidence interval
ROC: Receiver operating characteristic
AUC: Area under curve
